# Establishment of Pathogen-Free *Rhipicephalus bursa* Colonies Under Laboratory Conditions for the Vector Competence Studies

**DOI:** 10.3390/vetsci12010054

**Published:** 2025-01-13

**Authors:** Mehmet Can Ulucesme, Sezayi Ozubek, Munir Aktas

**Affiliations:** Department of Parasitology, Faculty of Veterinary Medicine, University of Fırat, Elazığ 23200, Türkiye; sozubek@firat.edu.tr (S.O.); maktas@firat.edu.tr (M.A.)

**Keywords:** *Rhipicephalus bursa*, tick colony, tick-borne pathogen-free, vector competence study

## Abstract

*Rhipicephalus bursa*, the primary vector of *Babesia ovis*, is also considered to transmit *Theileria*, *Babesia*, and *Anaplasma* spp. These claims are based on pathogen detections rather than experimental validation. To confirm vector competence, sterile ticks must acquire pathogens from infected hosts and transmit them to other hosts. The basic step is establishing a pathogen-free tick colony. In this study, engorged *R. bursa* females were collected from 12 infested livestock and allowed to lay eggs. The carcasses and larvae were screened for tick-borne pathogens using nPCR. The 0.150 g pathogen-free F1 larvae were fed on New Zealand rabbits, resulting in 592 engorged nymphs that molted into F1 adults. Eighty F1 adults were fed on pathogen-free splenectomized sheep, producing the next larval generation (F2). This protocol was repeated to produce F3 larvae. At the end of all developmental stages, ticks were screened via nPCR and found to be negative for tick-borne pathogens. The sheep were monitored for 63 days with no clinical signs or positive nPCR results, confirming F3 larvae as pathogen-free and suitable for vector competence studies. The *R. bursa* life cycle was completed in 72–153 days, providing a reliable model for vector competence research and offering valuable insights into its biological parameters.

## Simple Summary

In this study, pathogen-free *Rhipicephalus bursa* colonies were established for vector competence research, and their life cycle parameters were analyzed. Initially, engorged *R. bursa* females were collected from naturally infested sheep, goats, and cattle. The engorged females were placed in an incubator to lay eggs and produce larvae (F1 larvae). Two New Zealand rabbits and two pathogen-free splenectomized sheep were used to obtain subsequent generations. The F1 and F2 unfed larvae were fed on rabbits, and F1 and F2 unfed adults were fed on sheep. F3 larvae were obtained from engorged F2 females. At the end of all developmental stages, tick pools were screened using nPCR for tick-borne pathogens and found to be pathogen-free. The sheep were clinically monitored for 63 days, during which no clinical signs of disease were observed, and all tests for the presence of tick-borne pathogens yielded negative results. F3 larvae were confirmed as pathogen-free and suitable for vector competence studies. Under laboratory conditions, the *R. bursa* life cycle was completed in 72–153 days. This study demonstrated that pathogen-free *R. bursa* colonies can be maintained over multiple generations, offering a reliable model for vector competence research. Establishing these colonies and documenting their biological parameters is crucial for advancing strategies in vector-borne disease control.

## 1. Introduction

Ticks are obligate hematophagous ectoparasites that transmit numerous significant pathogenic microorganisms [1,2,3,4]. Ticks (Acari: Ixodidae) are known to infest a wide range of hosts, including humans, livestock, pets, wildlife, reptiles, and birds, and are the second most common arthropod vector for disease transmission after mosquitoes [5,6]. Tick-borne diseases (TBDs) such as theileriosis, babesiosis, anaplasmosis, and ehrlichiosis are critical health challenges for small ruminants in tropical and subtropical regions [7,8,9,10,11,12,13,14].

Among TBDs, theileriosis, babesiosis, and anaplasmosis are notable for their impact on sheep and goats. These diseases are transmitted primarily by *Hyalomma*, *Rhipicephalus,* and *Haemaphysalis* ticks [15,16,17,18,19,20,21,22]. *Theileria lestequardi*, a pathogenic agent causing theileriosis in small ruminants, is known to be transmitted by *Hyalomma* spp.; however, *R. bursa* is also suggested as a potential vector [23]. Although typically non-pathogenic, *T. ovis* is commonly found in *R. bursa* and other *Rhipicephalus* species feeding on sheep and goats [24,25]. *Rhipicephalus bursa*, a two-host tick widely distributed throughout the Mediterranean region, is the primary vector associated with *Babesia ovis*, in which it transmits through both transtadial and transovarial routes [7,19,21,26,27,28]. However, *R. bursa* lacks vector competence for *Babesia aktasi*, a newly identified species infecting goats [29]. *Babesia motasi* is exclusively transmitted by ticks of the *Haemaphysalis* genus [22]. Given its widespread presence and vector potential in small ruminants, understanding the role of *R. bursa* in transmitting various tick-borne pathogens remains crucial for disease management in these animals.

The developmental stages of *R. bursa* have seasonal activity. It has been stated that the larvae of *R. bursa*, which complete one generation per year under natural conditions, are most active during the winter months, and the adult stage is most active during the summer months when temperatures increase [30,31]. The high incidence of babesiosis caused by *B. ovis* in sheep and goats during the summer months has been associated with the activeness of *R. bursa* adults during this period. However, this process exhibits a life cycle ranging from 99 to 214 days under laboratory conditions [19,21,26,31,32,33]. Both the immature and adult stages of *R. bursa* infest sheep, goats, cattle, horses, donkeys, and rarely, wild ungulates, although the preferred hosts are sheep and goats [31,34,35].

Accurate determination of vector competence is vital for understanding the epidemiology of tick-borne pathogens. Many reports rely on the co-occurrence of ticks and diseases, rather than experimental infection studies, to infer transmission, which can complicate epidemiological understanding and lead to errors in identifying vector-parasite relationships [36]. Detecting pathogens’ DNA in ticks alone is insufficient to confirm vector competence [37,38]. In ixodid ticks, the terms “vectorial capacity” and “vector competence” are concepts commonly used to describe an arthropod’s ability to act as a vector for a pathogen [39]. The vectorial capacity of a tick can be recognized if the existing epidemiological relationship (for example, the correlation between tick presence and disease occurrence) is experimentally supported by tick transmission studies [36]. Elucidating the mechanisms involved in tick–pathogen interactions that influence vector competence is essential for identifying the molecular drivers of tick-borne diseases, which in turn, facilitates the development of paradigms for disease control and prevention [40]. Identifying which tick species transmits a specific pathogen is reported to be crucial for understanding and studying the biology of that pathogen [37]. To determine whether a tick has vector competence for any blood parasite, one of the most established experimental approaches involves allowing sterile ticks to feed on an experimentally infected host (or infected blood in in vitro experiments) to acquire TBP forms. Before starting experimental tick transmission studies, the fundamental step is establishing a sterile laboratory colony of ticks to be used in the experiments [37,41].

Understanding the life cycle of ticks is essential for studying tick-borne pathogens and developing effective control strategies. This study focuses on the development of a pathogen-free *R. bursa* colony under controlled laboratory conditions, a prerequisite for experimental vector competence research. We examined the life cycle of *R. bursa*, including its developmental stages, feeding behavior, and pathogen acquisition potential, to establish a reliable model for studying the transmission dynamics of tick-borne pathogens.

## 2. Materials and Methods

### 2.1. Tick Collection, Identification, and Molecular Screening for Tick-Borne Pathogens

**Figure 1:**
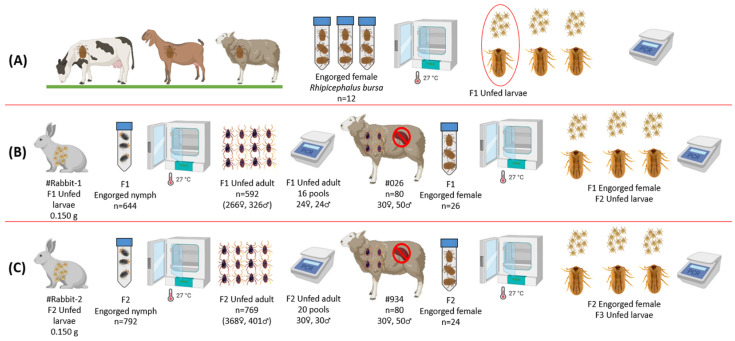
The representative scheme of the study design. (**A**) Collecting engorged ticks from cattle, sheep, and goats naturally infested with ticks, oviposition of *R. bursa* engorged female adults, hatching larvae, and the determination of pathogen-free (*Babesia*, *Theileria*, *Anaplasma*, and *Ehrlichia* spp.) larval bathes. (**B**,**C**) Infestation of the rabbits and splenectomized sheep by immature and adult stages of *R. bursa,* respectively, and screening by nPCR for the detection of *Babesia* spp., *Theileria* spp., *Anaplasma* spp., and *Ehrlichia* spp. Figure 1 was created using BioRender.com (https://www.biorender.com/ accessed on 14 November 2024).

In the surrounding villages of Elazığ province in eastern Türkiye, 19 apparently healthy animals (13 sheep, 4 goats, and 2 cattle) grazing outdoors were examined for tick infestations in May 2021. A total of 327 ticks were collected from these animals and brought to the Department of Parasitology at the Veterinary Faculty of Fırat University for species identification. Each tick’s sex was recorded, and species identification was performed using a stereomicroscope and a taxonomic key [42]. Blood samples (2 mL each) were collected from the jugular vein of each animal and preserved in EDTA tubes. Among the collected ticks, 12 engorged female ticks identified as *Rhipicephalus bursa* were kept in an incubator set to 27 ± 1 °C and 70–80% relative humidity (RH) for egg-laying and hatching of larvae. After oviposition, 12 engorged female tick carcasses were removed from the incubator, bisected longitudinally with a sterile scalpel, and stored at −20 °C for DNA extraction. Four larvae pools (each containing approximately 100 larvae) were obtained from larval batches belonging to each engorged female tick. Engorged female tick carcasses and the larval pools from each engorged female tick were crushed using sterile plastic rods in liquid nitrogen and prepared for genomic DNA extraction [20,43]. The representative scheme of the experimental study is presented in Figure 1.

Genomic DNA was extracted using a PureLink™ Genomic DNA Mini Kit (Invitrogen, Invitrogen, Carlsbad, CA, USA), and nPCR was performed for *Babesia*, *Theileria*, *Anaplasma*, and *Ehrlichia* species using specific primers [44,45,46,47,48] (Supplementary Table S1). Positive (*B. ovis and A. ovis* genomic DNA from GenBank accession numbers no.EF092454.1 and no.MG693754.1, respectively) and negative (DNase/RNase-free water) controls were included in each PCR reaction. Reactions were conducted in an automated DNA Sensequest thermal cycler (Labcycler Gradient, Göttingen, Germany). Ten microliters of the PCR products were electrophoresed on a 1.4% agarose gel for 30 min and visualized using the Quantum Vilber Lourmat (Marne-la-Vallee, France) gel imaging system. To verify the DNA of *R. bursa*, PCR was conducted using 16S  +  1 and 16S − 1 primers [49] (Supplementary Figure S1).

### 2.2. Feeding of R. bursa Larvae on Rabbits

Two larval batches from engorged female ticks (#202 and #212) confirmed to be pathogen-free by nPCR were used for rabbit infestation. Approximately 0.150 g of active larvae from sheep #212 (F1 larvae) were placed in EVA foam capsules attached to a New Zealand rabbit (#Rabbit-1) as described by Almazan et al. [50]. Engorged nymphs were collected daily, incubated at 27 ± 1 °C and 70 ± 10% RH, and allowed to molt into F1 unfed adults. DNA extracted from 16 pools of F1 unfed adults (8 male, 8 female, and 3 ticks per pool) was screened by nPCR to confirm pathogen-free status.

### 2.3. Feeding of R. bursa Adult on Sheep

Two splenectomized sheep (#026 and #934), confirmed to be pathogen-free by nPCR and microscopy, were used to feed unfed *R. bursa* adults. Splenectomy was performed at the Fırat University animal hospital following standard anesthesia, analgesia, and aseptic procedures [51,52]. After surgery, sheep were monitored for three weeks for clinical signs and retested for *Theileria*, *Babesia*, and *Anaplasma* spp.

EVA foam feeding capsules were attached to the thoracic region of sheep #026, and 80 F1 unfed adults (30 females, 50 males) were introduced. Engorged females were collected, incubated for oviposition, and their carcasses, along with F2 larval pools, were screened by nPCR. F2 larvae were fed on Rabbit-2, and the resulting F2 adults were fed on sheep #934, following the same procedures. Sheep #026 and #934 were monitored clinically and by nPCR for 63 days post-infestation.

F2 engorged females from sheep #934 were incubated for oviposition, generating F3 larvae. Both F2 female carcasses and F3 larval pools were screened for *Theileria*, *Babesia*, and *Anaplasma* spp. to confirm a pathogen-free status before subsequent use.

### 2.4. Assessing the Development and Biological Parameters of Immature and Adult R. bursa

During the completion of their life cycles, the feeding periods of *R. bursa* larvae on rabbits and adults on sheep were carefully monitored. Key biological parameters were evaluated, including the weight of engorged females, the duration of the pre-oviposition period, oviposition period, egg incubation period, and molting period.

## 3. Results

In this study, a total of 327 adult ticks (87 females and 240 males) were collected from 19 animals, consisting of 13 sheep, 4 goats, and 2 cattle. Morphological identification revealed three tick species: *Rhipicephalus bursa* (39 females and 96 males), *R. turanicus* (37 females and 100 males), and *H. marginatum* (11 females and 44 males) (Table 1). Among the 39 female *R. bursa*, 12 were fully engorged and suitable for oviposition (Table 2).

**Figure 2:**
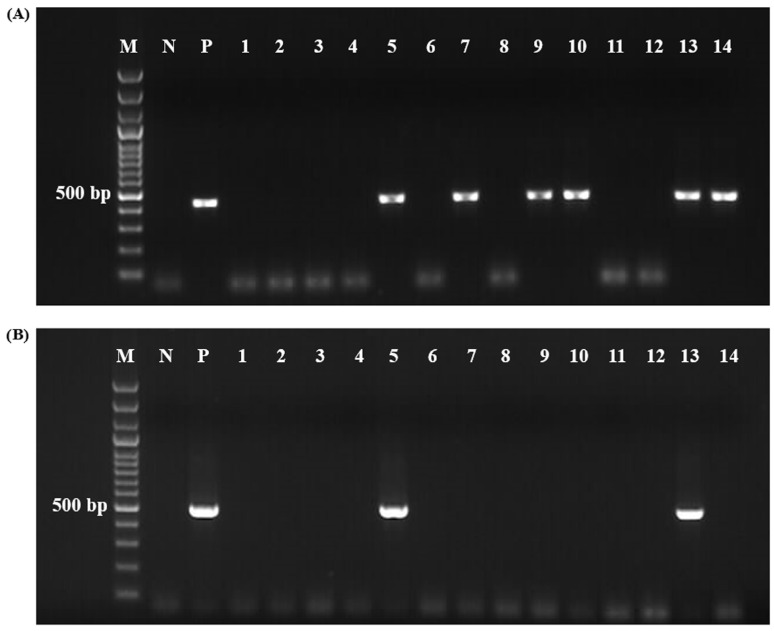
Gel imaging of nPCR results of carcasses and larvae from engorged *R. bursa* females collected from animals. (**A**) Gel image showing positive and negative nPCR amplification products representing *Babesia* and *Theileria* species obtained using Nbab1F/Nbab1R [47] and RLB-F2/RLB-R2 [48] primers. M: 100 bp marker, N: negative control (distilled water), P: *Babesia ovis* (EF092454), Lanes 1, 3, 5, 7, 9, 11, and 13 show DNA from engorged *R. bursa* females (#202-1, #204-1, #207-1, #209-1, #211-1, #212-1, and #216-1, respectively). Lanes 2, 4, 6, 8, 10, 12, and 14 represent their corresponding larval pools. (**B**) Gel image showing positive and negative nPCR amplification products representing *Anaplasma* and *Ehrlichia* species obtained using Ec9/Ec12a [45] and 16S8FE/BGA1B [46] primers. P: *Anaplasma ovis* (MG693754). Sample order is identical to Panel A.

After incubation, the 12 engorged *R. bursa* females initiated oviposition within 4–7 days. The first larvae began hatching between days 29 and 33 post oviposition, with larval hatching completing within 5–14 days. Nested PCR results indicated that only 2 of the 12 engorged female carcasses, along with their larval pools (#202 and #212) tested negative for *Theileria*, *Babesia*, *Anaplasma,* and *Ehrlichia* spp. (Figure 2). Additionally, the blood samples from the host animals from which these two engorged females were also negative for these tick-borne pathogens. In contrast, the remaining host blood samples, tick carcasses, and larval pools tested positive by nPCR (Table 2).

Based on nPCR analysis results, larval pools #202 and #212, which were negative, were selected. The more active larval batch (F1 larvae) from pool #212 was used to feed on #Rabbit-1. Approximately 0.150 g of F1 unfed larvae were placed in a capsule attached to #Rabbit-1 and allowed for around 18 days. Following the feeding period, 644 engorged nymphs were collected from the capsule 29 days after initial attachment. Of these, 592 nymphs (91.9%) successfully molted into adult ticks (F1 unfed adults), comprising 266 females and 326 males (Table 3). In 4 days, 345 engorged nymphs dropped, producing 286 (82.6%) male adults and 60 (17.4%) female adults. Sixteen pools of adult ticks, with each pool containing three F1 unfed adults (48 unfed adults) were screened using nPCR. The results indicated that all pools were negative for *Theileria*, *Babesia*, *Anaplasma,* and *Ehrlichia* spp.

Nested PCR-negative thirty F1 unfed female adults were attached to splenectomized sheep #026 for feeding, and 26 of them had fed and become engorged females after 6–10 days. The heaviest engorged female weighed 0.673 g on day 8, producing an egg mass of 0.348 g after detachment from the sheep. Engorged females that separated from the egg mass survived up to 4 days. Approximately 30 days after the oviposition period, next generation-larvae (F2 unfed larvae) were obtained from the engorged females (Table 4). All fifty-two pools and the carcasses of the engorged females were screened by nPCR for *Theileria*, *Babesia*, and *Anaplasma* spp. with all samples testing negative (Table 5).

**Figure 3:**
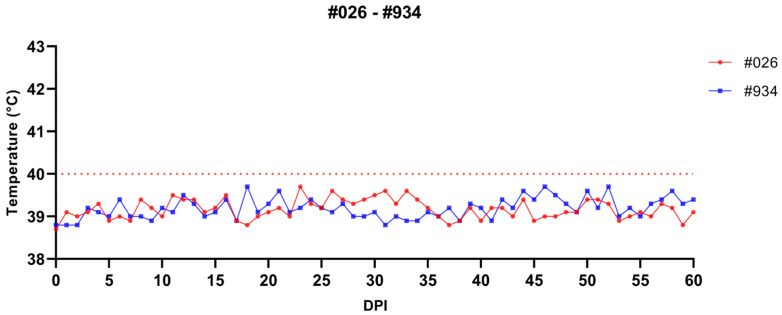
Monitoring body temperature of splenectomized sheep (#026 and #934) infested with *R. bursa* unfed adults (F1 and F2 generations). The red line (40 °C) represents the maximum body temperature observed in healthy sheep. DPI: Day post infestation.

**Figure 4:**
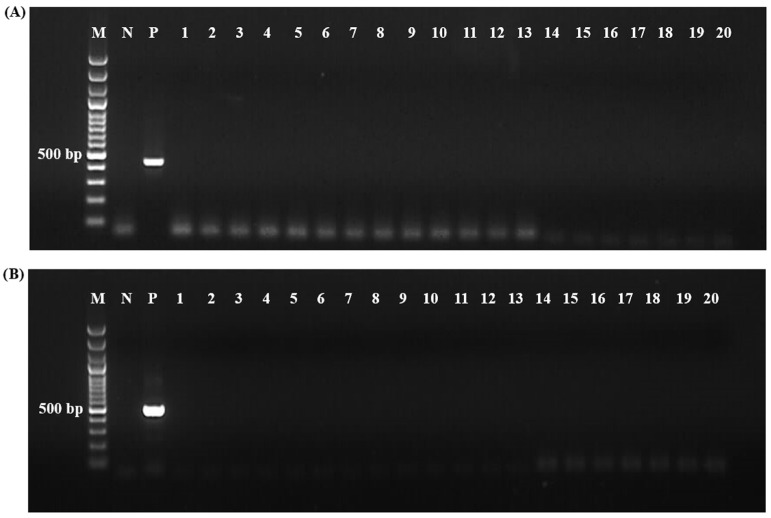
Nested PCR analysis of *Babesia* and *Theileria* (Panel **A**) and *Anaplasma* and *Ehrlichia* (Panel **B**) in splenectomized sheep #026 and #934 up to 63 days post-infestation. All results were negative for tick-borne pathogens after infestation with F1 and F2 unfed R. bursa adult ticks.

The splenectomized sheep #026 and #934 used in the experimental period showed no clinical signs of tick-borne diseases for 63 days. Body temperature remained within the normal range in both sheep (Figure 3), and nPCR analysis results were negative for both animals (Figure 4).

## 4. Discussion

*Rhipicephalus bursa* is a two-host tick species primarily associated with the transmission of *B. ovis* and other pathogens that impact livestock health, particularly in the Mediterranean and Middle Eastern regions [19,26]. This tick species completes its life cycle over one generation per year and has a unique developmental pattern where both the immature (larval and nymph) and adult stages parasitize similar primary hosts, including cattle, sheep, and goats [21].

In this study, we aimed to obtain colonies of different stages of new-generation tick-borne pathogen-free *R. bursa* for use in vector competence studies. Our findings indicated that the life cycle of *R. bursa* from preoviposition to the next-generation engorged adult female was completed within 72–153 days. This is notably shorter than the 99–254 days reported by Yeruham et al. [33] for *R. bursa* when completing all developmental stages on lambs. The observed difference is likely due to variations in pre-molting and molting periods of engorged nymphs, which may result from feeding on different host species. Additionally, the three-host tick *R. sanguineus* completes its life cycle in 70–126 days [53], while another study reported this duration to be between 162 and 177 days [54]. The shorter duration observed in our study compared to previous reports may reflect specific environmental conditions under which the ticks were maintained, such as controlled laboratory settings or differences in strain adaptability. These findings underscore the importance of considering host, environmental, and strain-specific factors when interpreting the life cycle dynamics of *R. bursa* and other tick species [53,55].

In this study, the preoviposition period showed similarity to findings reported in previous research [33,56]. Specifically, the mean preoviposition period of engorged females dropped from sheep #026 was 5.1 days, matching the value reported by Yeruham et al. [33]. Comparatively, the preoviposition period for *R. sanguineus* and *R. turanicus* have been recorded as 5.9 and 4.5 days, respectively [57]. These slight variations among *Rhipicephalus* likely reflect differences in metabolic rates and the female’s ability to convert body protein content into eggs, independent of feeding [58]. In this study, the oviposition period was determined to be 12.4 and 13.1 days, which is shorter than the 16.5 ± 2.44 days reported by Yeruham et al. [33]. This difference may be attributed to variations in the feeding period and related physiological factors [58,59,60]. Additionally, the duration from unfed adult to engorged female averaged 9 days in our study, whereas Yeruham et al. [33] reported a shorter duration of 5.9 days. These findings suggested an inverse correlation between blood-feeding duration and subsequent reproductive parameters, emphasizing the influence of host–tick interactions and environmental conditions on tick development.

In this study, a positive correlation was found between the weight of engorged *R. bursa* females and the weight of the eggs laid. Approximately 50.9% of the engorged female’s weight was converted into egg mass, consistent with the findings of Yeruham et al. [33]. Similar relationships have been reported in other *Rhipicephalus* species [53], as well as in the species of the *Dermacentor* spp. [61] and *Hyalomma* spp. [62], highlighting the conserved nature of this reproductive efficiency across different tick species. The incubation period for *R. bursa* eggs until larval hatching was determined to be 28.9 and 31.8 days, aligning with previous findings [33]. This period was also similar to that reported for *R. sanguineus* [53]. It is well established that egg incubation and hatching periods are influenced by environmental factors such as temperature [63], which could explain minor variations observed across studies.

In the study, the weights of the engorged nymphs were not measured; however, based on external observation, it was found that the majority of the early-dropping engorged nymphs with lower weights produced male adults. On the other hand, the engorged nymphs that fed for a longer period predominantly produced female adults. In the study by Yeruham et al. [33], it was reported that 76.1% of nymphs weighing up to 8.9 mg produced male adults. Nymphs that detached in the first half of the drop-off phase tended to produce males, while those that detached in the second half tended to produce females. Our findings further supported these observations, with 88.2% and 82.6% of nymphs producing male adults. These results emphasize the significant role of feeding duration and body weight in influencing sex determination in *R. bursa*.

Before and after being placed on the host, larvae and adults from different generations were negative for *Theileria*, *Babesia*, *Anaplasma*, and *Ehrlichia* spp. through nPCR. The sheep on which the adult ticks fed were monitored clinically and molecularly for 63 days. Clinically, no symptoms related to tick-borne pathogens were observed, and nPCR confirmed their negative status. Based on these findings, the F3 *R.bursa* unfed larvae obtained from the laboratory colonies were considered sterile, and it was decided to use this colony in vector studies. These results provide strong evidence that the laboratory-produced colonies of *R. bursa* are free of pathogens and can be safely utilized in vector-related research. Nevertheless, this study is limited by the relatively small sample size of ticks and animals used, which may affect the generalizability of the findings. Larger-scale studies involving more extensive tick and host populations would provide a more comprehensive understanding of the life cycle dynamics and host-tick interactions. Additionally, future research could explore genetic and environmental factors influencing tick development to further elucidate the biological and ecological characteristics of *R. bursa*.

## 5. Conclusions

This study provided valuable insights into the life cycle dynamics and reproductive parameters of *R. bursa*, emphasizing its importance as a vector of *B. ovis* and other livestock pathogens in the Mediterranean and Middle Eastern regions. The results demonstrated that laboratory-produced colonies of *R. bursa* can be successfully maintained pathogen-free across multiple generations, offering a reliable model for vector competence studies. Critical parameters such as preoviposition and oviposition periods, egg incubation duration, and sex determination based on nymph feeding behaviors were found to align closely with previous studies, highlighting the consistency of *R. bursa* biology across different environments. However, the study’s small sample size is a limitation that underscores the need for larger-scale studies to confirm these findings and improve the generalizability of the results. Future investigations should also focus on the genetic and environmental factors that influence tick development and reproductive efficiency. The establishment of pathogen-free tick colonies also underscores the feasibility of developing targeted strategies for controlling vector populations in livestock settings. Given the significant role of *R. bursa* in transmitting *B. ovis* and other pathogens, understanding its vectorial capacity is crucial for mitigating tick-borne diseases. The findings of this study highlight the urgent need to develop specific vaccines and preventative measures against *R. bursa*, as such advancements are essential for protecting livestock health and ensuring sustainable animal production systems.

## Figures and Tables

**Table 1:** Number of collected ixodid tick species from sheep, goat, and cattle in the field.

Animal ID	Host	Collected Tick Species					
		*R. bursa*		*R. turanicus*		*H. marginatum*	
		Female (♀)	Male (♂)	Female (♀)	Male (♂)	Female (♀)	Male (♂)
#201	Sheep	1	1	-	-	-	-
#202	Sheep	1	3	2	8	-	-
#203	Goat	3	4	4	6	-	-
#204	Sheep	5	14	-	-	3	11
#205	Goat	1	1	-	4	-	-
#206	Sheep	2	2	5	4	-	-
#207	Sheep	3	4	1	1	-	-
#208	Goat	1	3	2	3	-	-
#209	Sheep	3	7	-	-	1	1
#210	Sheep	2	5	3	17	-	1
#211	Sheep	2	2	-	-	-	-
#212	Sheep	2	6	1	5	-	-
#213	Sheep	-	2	3	3	-	-
#214	Goat	-	-	1	3	-	-
#215	Sheep	3	18	3	21	3	2
#216	Sheep	6	16	4	14	1	6
#217	Sheep	2	2	1	1	-	-
#218	Cattle	-	-	4	6	3	18
#219	Cattle	2	6	3	4	-	5
Total		39	96	37	100	11	44

**Table 2:** Nested PCR results for *R. bursa* engorged females, carcasses, larval pools, and host blood samples for detection of *Theileria*, *Babesia*, *Anaplasma*, and *Ehrlichia* spp.

Animal ID	*R. bursa*		nPCR Results					
	Number of Engorged Female *	Carcass ID	*Theileria*/*Babesia* spp.			*Anaplasma*/*Ehrlichia* spp.		
			Blood	Carcass	Larvae Pools	Blood	Carcass	Larvae Pools
#202	1 *	#202-1	-	-	-	-	-	-
#204	1	#204-1	+	-	-	-	-	-
#207	2	#207-1	+	+	-	+	+	-
		#207-2		+	-		+	-
#209	1	#209-1	+	+	-	-	-	-
#211	1	#211-1	+	+	+	-	-	-
#212	2 *	#212-1	-	-	-	-	-	-
		#212-2		-	-	-	-	-
#216	4	#216-1	+	+	+	+	+	-
		#216-2		+	+		+	-
		#216-3		+	+		+	-
		#216-4		+	+		+	-

* Number of engorged females kept in an incubator for oviposition and larval hatching.

**Table 3:** Life cycle and biological parameters of immature *R. bursa* fed on New Zealand rabbits under laboratory conditions.

Life Stage	Parameters	Number of Days			
		Rabbit-1		Rabbit-2	
		Min-Max	Mean	Min-Max	Mean
Larva	Pre-feeding period	1–3	2	1–4	2.5
	Feeding period	13–20	18.1	14–22	19.2
Engorged nymph	Dropp-off period	7	7	8	8
	Incubation and molting period	12–29	19.2	13–22	21

**Table 4:** Developmental parameters of engorged female ticks collected from sheep #026 and #934, including oviposition, egg mass, and larval development periods.

Life Stage	Parameters	#026		#934	
		Min–Max	Mean	Min–Max	Mean
Engorged female	Engorged females weight (g)	0.362–0.673	0.558	0.139–0.747	0.550
	Pre-oviposition (days)	2–7	5.1	3–8	5.6
	Oviposition period (days)	7–17	12.4	6–18	13.1
	Post-oviposition survival (days)	1–4	1.5	1–4	1.4
Eggs	Eggs mass weight (g)	0.165–0.348	0.284	0.04–0.358	0.258
	Incubation period (days)	30–36	31.8	21–39	28.9
Larva	Larval hatching period (days)	4–13	7.4	7–13	7.8

(g; gram).

**Table 5:** Nested PCR results *of Rhipicephalus bursa* obtained at different generations and developmental stages.

Tick Stage	Generation	No. of Ticks in Each Pool	No. of Pools	No. of Total Ticks	No. nPCR-Positive Ticks/No. Pools Tested
Unfed larvae	F1	100	4	400	0/4
Unfed adult	F1	3	16	48	0/16
Engorged adult *	F1			26	0/26 *
Unfed larvae	F2	100	52	5200	0/52
Unfed adult	F2	3	20	60	0/20
Engorged adult *	F2			24	0/24 *
Unfed larvae	F3	100	48	4800	0/48

* Individual tick sample.

## Data Availability

The original contributions presented in the study are included in the article, further inquiries can be directed at the corresponding author.

## Supplementary Materials

The following supporting information can be downloaded at: https://www.mdpi.com/article/10.3390/vetsci12010054/s1, Figure S1: Unfed larva, male, and female *Rhipicephalus bursa* from colonies used in the study: (A) Larvae, (B) Male, and (C) Female. Scale bar: 0.985 mm. Figure S2: Experimental design and infestation process for *Rhipicephalus bursa*: (A) Infestation of rabbits with the immature stages (larvae and nymphs) of *R. bursa* using EVA foam, followed by the collection of engorged nymphs. (B) Infestation of sheep with adult *R. bursa*, followed by the collection of engorged females. Figure S3: Verification of *Rhipicephalus bursa* DNA using PCR with 16S  +  1 and 16S – 1 primers. Lane N: Negative control (DNase/RNase-free water); Lane 1: F1 unfed larvae; Lane 2: F1 unfed adults; Lane 3: F2 unfed larvae; Lane 4: F2 unfed adults; and Lane 5: F3 unfed larvae. Table S1: Primers and sequences used in this study for the detection of tick-borne pathogens.
