# Supplementary material for: Establishment of Pathogen-Free Rhipicephalus bursa Colonies Under Laboratory Conditions for the Vector Competence Studies
Source: Vet Sci. 2025 Jan 13;12(1):54. doi: 10.3390/vetsci12010054 (PMC11768552; doi:10.3390/vetsci12010054)
Supplement: Supplementary file 1 [file vetsci-12-00054-s001.zip › Table S1.pdf]

Table S1: Primers and sequences used in this study for the detection of tick-borne pathogens

Tick-borne pathogens		Target gene	Primer	Primer sequence (5′–‘3)	Reference
Anaplasma / Ehrlichia	1st reaction	*16S rDNA*	Ec9	TACCTTGTTACGACTT	[45]
			Ec12A	TGATCCTGGCTCAGAACGAACG	
	nPCR		16S8FE	GGAATTCAGAGTTGGATC(A/C)TGG(C/T)TCAG	[46]
			BGA1B	CGGGATCCCGAGTTTGCCGGGACTT(C/T)TTCT	
Babesia /Theileria	1st reaction	*18S rDNA*	Nbab1F	AAGCCATGCATGTCTAAGTATAAGCTTTT	[47]
			Nbab1R	CTTCTCCTTCCTTTAAGTGATAAGGTTCAC	
	nPCR		RLBF2	GACACAGGGAGGTAGTGACAAG	[48]
			RLBR2	CTAAGAATTTCACCTCTGACAGT	
