# Supplementary figures and images for: Establishment of Pathogen-Free Rhipicephalus bursa Colonies Under Laboratory Conditions for the Vector Competence Studies
Source: Vet Sci. 2025 Jan 13;12(1):54. doi: 10.3390/vetsci12010054 (PMC11768552; doi:10.3390/vetsci12010054)

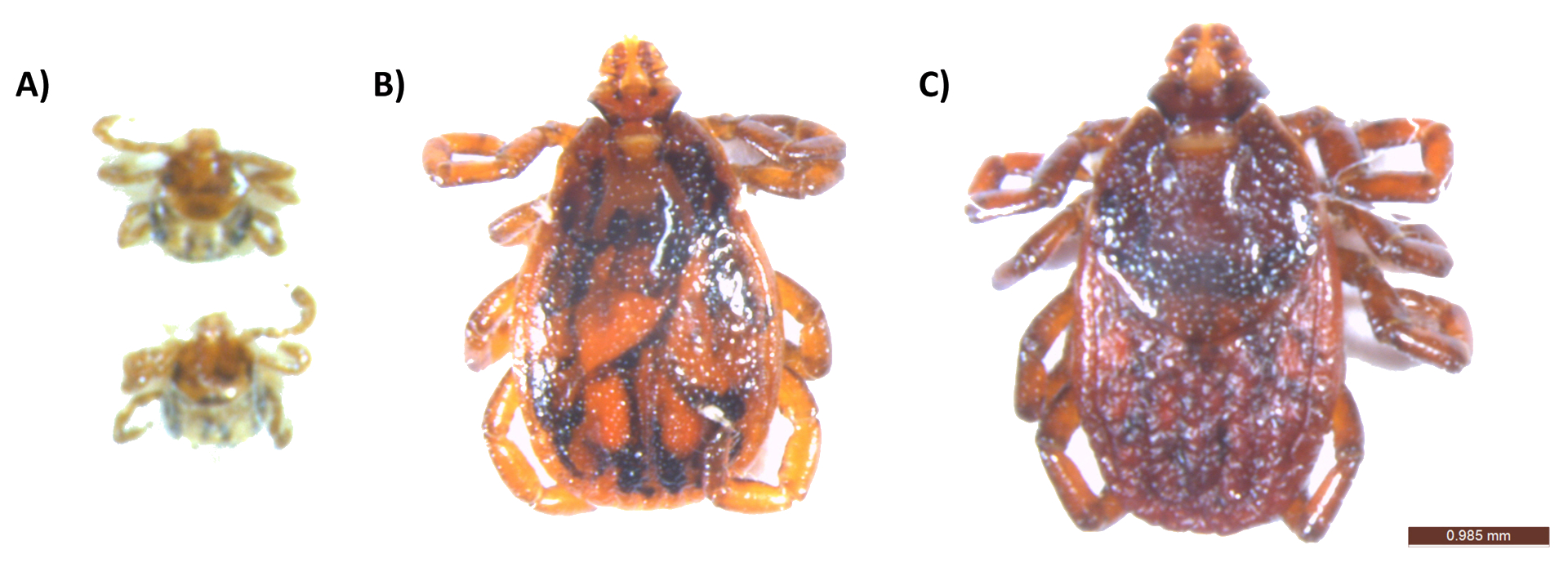

Supplement: Supplementary file 1 [file vetsci-12-00054-s001.zip › Figure S1.tif]

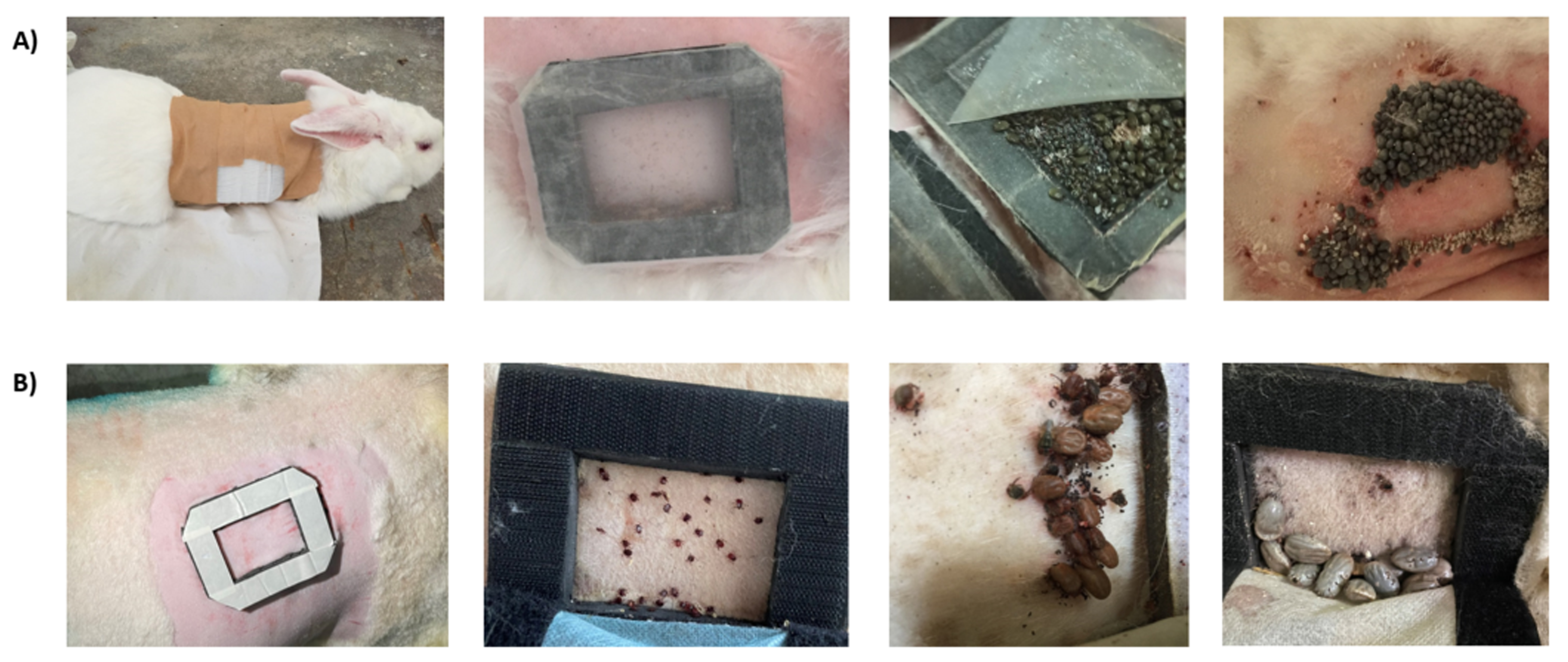

Supplement: Supplementary file 1 [file vetsci-12-00054-s001.zip › Figure S2.tif]

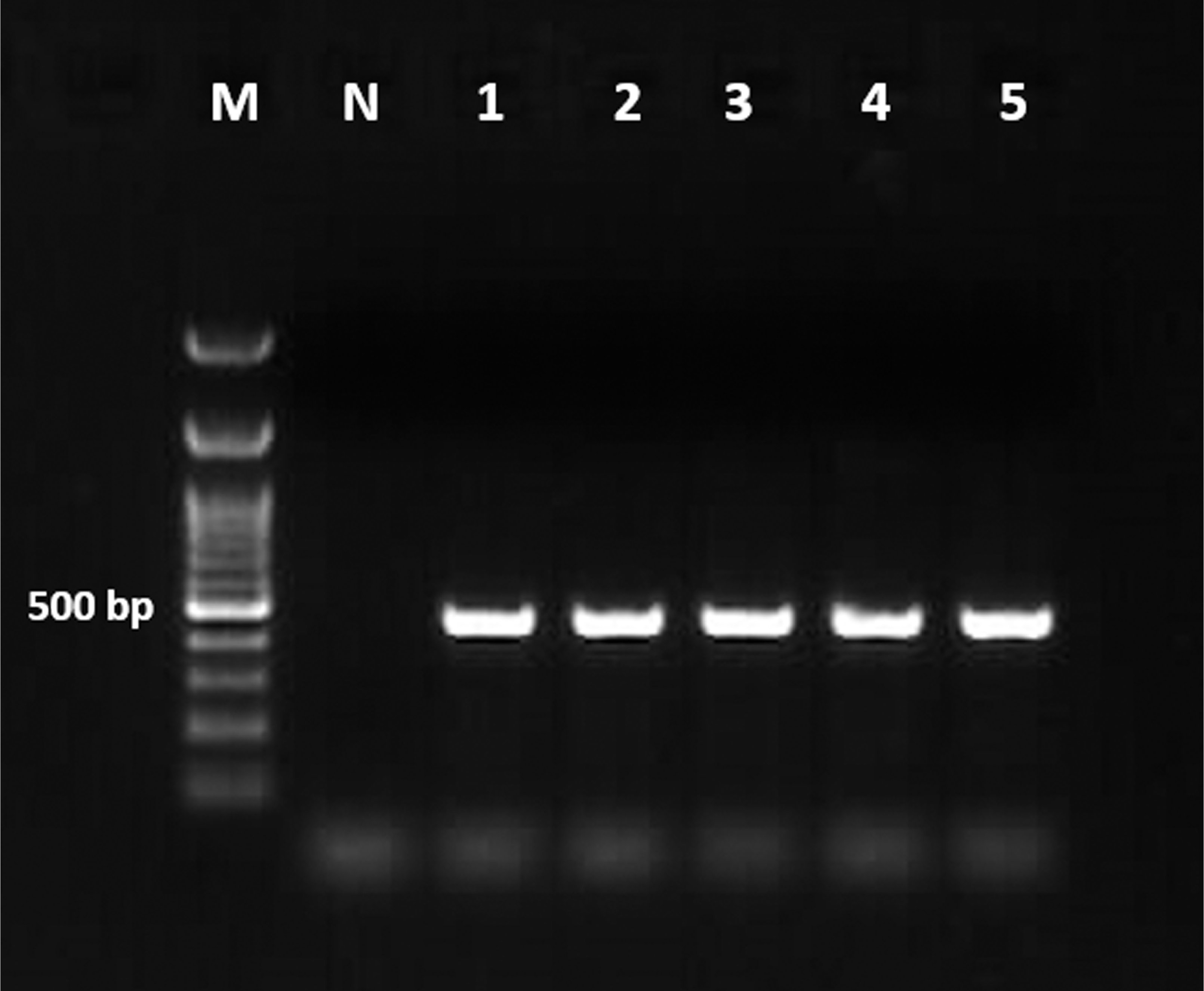

Supplement: Supplementary file 1 [file vetsci-12-00054-s001.zip › Figure S3.tif]
